# YAP signaling orchestrates the endothelin-1-guided invadopodia formation in high-grade serous ovarian cancer

**DOI:** 10.1042/BSR20241320

**Published:** 2024-11-29

**Authors:** Piera Tocci, Valentina Caprara, Celia Roman, Rosanna Sestito, Laura Rosanò, Anna Bagnato

**Affiliations:** 1 Preclinical Models and New Therapeutic Agents Unit, Istituto di Ricovero e Cura a Carattere Scientifico (IRCCS), Regina Elena National Cancer Institute, Rome, Italy; 2 Institute of Molecular Biology and Pathology (IBPM), National Research Council (CNR), Rome 00185, Italy

**Keywords:** endothelin-1 receptor, invadopodia, metastasis, ovarian cancer, YAP

## Abstract

The high-grade serous ovarian cancer (HG-SOC) is a notoriously challenging disease, characterized by a rapid peritoneal dissemination. HG-SOC cells leverage actin-rich membrane protrusions, known as invadopodia, to degrade the surrounding extracellular matrix (ECM) and invade, initiating the metastatic cascade. In HG-SOC, the endothelin-1 (ET-1)/endothelin A receptor (ET_A_R)-driven signaling coordinates invadopodia activity, however how this axis integrates pro-oncogenic signaling routes, as YAP-driven one, impacting on the invadopodia-mediated ECM degradation and metastatic progression, deserves a deeper investigation. Herein, we observed that downstream of the ET-1/ET-1R axis, the RhoC and Rac1 GTPases, acting as signaling intermediaries, promote the de-phosphorylation and nuclear accumulation of YAP. Conversely, the treatment with the dual ET_A_/ET_B_ receptor antagonist, macitentan, inhibits the ET-1-driven YAP activity. Similarly, RhoC silencing, or cell transfection with a dominant inactive form of Rac1, restores YAP phosphorylation. Mechanistically, the ET-1R/YAP signal alliance coordinates invadopodia maturation into ECM-degrading structures, indicating how such ET-1R-guided protein network represents a route able to enhance the HG-SOC invasive potential. At functional level, we found that the interconnection between the ET-1R/RhoC and YAP signals is required for MMP-2 and MMP-9 proteolytic functions, cell invasion, and cytoskeleton architecture changes, supporting the HG-SOC metastatic strength. In HG-SOC patient-derived xenografts (PDX) macitentan, turning-off the invadopodia regulators RhoC/YAP, halts the metastatic colonization. ET-1R targeting, hindering the YAP activity, weakens the invadopodia machinery, embodying a promising therapeutic avenue to prevent peritoneal dissemination in HG-SOC.

## Introduction

The high-grade serous ovarian cancer (HG-SOC) is an intrinsically aggressive and highly metastatic malignancy. The absence of specific symptoms and the lack of early screening tools lead to late-stage diagnosis, when metastasis has already occurred [1–3]. During intra-abdominal dissemination, HG-SOC cells adhere to the mesothelial extracellular matrix (ECM) and form invadopodia, which allows them to engender distant metastasis [4]. The predisposition to form invadopodia, cell protrusions consisting of F-actin core filaments and surrounding regulatory proteins, including ARP2/3, N-WASP, and cofilin able to degrade the ECM, frequently reflects the invasive degree of tumor cells, and represents a crucial event that dictates the rate and route of the HG-SOC metastatic journey [5–8].

Despite the central contribution of invadopodia in the metastatic process, disentangle the regulatory mechanism at the root of invadopodia formation and maturation is instrumental to better comprehend metastasis and uncover new vulnerabilities for cancer intervention.

In the last decades the impact of tumor-promoting factors on invadopodia formation and activity has been investigated, leading to the identification of common invadopodia-converging signaling pathways [5–15]. Into the plethora of the drivers of the metastatic process has been recognized the endothelin-1 (ET-1) [6,7]. In detail, in serous ovarian cancer cells ET-1, acting through the endothelin A receptor (ET_A_R), a member of the G protein couple receptor family, mediates the recruitment of multiple invadopodia-activating signaling pathways, including the Rho GTPases-mediates signals, coordinating invadopodia dynamics [11–15]. Into the fray of the master transcriptional determinants engaged in response to ET-1R activation is emerged YAP, whose transcriptional repertoire enables the HG-SOC invasive behavior and impacts on tumor cell communication with stromal neighboring cells, empowering essential attributes of tumor cells, as the ability to escape to therapeutic treatments [16–20]. A small number of previous studies have analysed the role of YAP in invadopodia formation; however, their findings are controversial. One study identified YAP as an inducer of invadopodia. In particular, YAP/TEAD transcriptional program actively contributes to the invadopodia dynamics [21]. In contrast, another one suggests that YAP inhibition enhances the expression levels of essential invadopodia components, favouring invadopodia formation and matrix degradation [22]. Thus, further investigation is required to better understand the role of YAP within invadopodia machinery.

In this study, we reveal a distinct mechanism in which ET-1/ET-1R axis is tightly intertwined with the oncogenic YAP signaling promoting the invadopodia formation and maturation process. Clinically significant, we examined the benefit produced by the ET-1R targeting that, interfering with YAP-mediated invadopodia machinery and metastatic cascade, may embody a more effective intervention perspective for metastatic HG-SOC patients.

## Materials and methods

### Cells and chemical compounds

Patient-derived (PD) HG-SOC cells were isolated from ascitic fluid of HG-SOC patients undergoing surgery for ovarian tumor by laparotomy or paracentesis at the Gynaecological Oncology of our Institute. This cell line is named PMOV10 where PM stands for Preclinical Models, OV stands for ovarian serous cancer, and # is the order in which the cell line was established. PMOV10 cells (*TP53* mutant R337T) closely recapitulate the genomic traits, the histopathology and the molecular features of the HG-SOC patient (stage III, age 69) [17]. The ascitic sample collection together with the relative clinical information were approved by the Regina Elena institutional review board (IRB) after HG-SOC patients gave written informed consent. Briefly, cells were harvested by centrifugation at 200 × g for 5 min at room temperature, resuspended in Dulbecco’s PBS, and then centrifuged through Ficoll-Histopaque 1077 (Sigma-Aldrich, St. Louis, Missouri, U.S.A.). Interface cells were washed in culture medium, and 5 × 10^6^ viable cells were seeded in 75 cm^2^ culture flasks, in RPMI 1640 (Gibco, Grovemont Cir, Gaithersburg, U.S.A.) containing 1% penicillin‒streptomycin and 10% fetal bovine serum. The purity of primary cultures was assessed by immunophenotyping with a panel of monoclonal Abs (including WT1, keratin 7, calretinin, and OCT-125) recognizing ovarian tumor-associated antigens by the alkaline phosphatase–peroxidase–antiperoxidase method.

In particular, for this study we utilized early passage PMOV10 cells, which recapitulate the HG-SOC features. PMOV10 cells were characterized for the copy number expression of ET-1, ET_A_R, and β-arr1. *TP53* gene sequencing of PMOV10 cells displayed a single nucleotide (C > G) germline missense mutation (R337T) [17].

Kuramochi cells (JCRB0098) were obtained from the Japanese Collection of Research Bioresources (JCRB) Cell Bank. Normal human lung fibroblasts (WI-38, CCL-75 ATCC) were cultured with Eagle’s minimum essential medium (EMEM) (30-2003 ATCC), supplemented with 10% FBS and 1% penicillin‒streptomycin. Cell lines were authenticated by STR profiling and regularly controlled for mycoplasma infection. ET-1 (Sigma-Aldrich, MO, U.S.A.) was used at a 100 nM final concentration. Macitentan (Selleckchem, U.K.), also called ACT-064992 or N-[5-(4-Bromophenyl)-6-[2-[(5-bromo-2-pyrimidinyl)oxy]ethoxy]-4-pyrimidinyl]-N′-propyl-sulfamide, added 30 min before ET-1 when administered in combination, was used at a 1 μM final concentration.

### Immunoblotting

Whole-cell lysates were obtained as reported [17] and were used for electrophoresis on SDS-PAGE gels. Bands with the protein of interest were detected by using the enhanced chemiluminescence (ECL) detection from Bio-Rad (CA, U.S.A.). The antibodies used for the study were as follows: anti-RhoC (cat. #ab180785, 1:1000), anti-RhoA, B, C (cat. #ab175328, 1:1000), and anti-Rac1 (cat. #ab155938, 1:1000) were from Abcam (Cambridge, U.K.). Anti-pYAP (S127) (cat. #13008S, 1:1000), anti-YAP (cat. #12395S, 1:1000), anti-pCofilin (S3) (cat. #3311, 1:1000), and anti-Cofilin (cat. #3311, 1:1000) were from Cell Signaling Technology (MA, U.S.A.). Anti-MMP-2 (cat. #sc-6838, 1:200), anti-MMP9 (cat. #sc-21733, 1:200), anti-Tubulin (cat. #sc-32293, 1:200), and anti-β-actin (cat. #sc-47778, 1:200) were from Santa Cruz Biotechnology (CA, U.S.A.).

### Ectopic expression and silencing experiments

YAP1 was knocked-down for 72 h using SMART Pool ON-TARGET plus siRNA (L-012200-00-0050, containing the following four siRNA: J-012200-05, J-012200-06, J-012200-07, and J-012200-08, targeting the following sequences respectively: GCAC-CUAUCACUCUCGAGA, UGAGAACAAUGACGACCAA, GGUCAGAGAUACU-UCUUAA, CCACCAAGCUAGAUAAAGA). RhoC was knocked-down for 72 h using SMART Pool ON-TARGET plus siRNA (L-008555-00-0050, containing the following four siRNA: J-008555-05, J-008555-06, J-008555-07, and J-008555-08, targeting the following sequences respectively: GAAAGAAGCUGGUGAUCGU, GAACUAUAUUGCGGACAUU, GGACAUGGCGAACCGGAUC, CUACGUCCCUACUGUCUUU) (Dharmacon RNA Technology, CO, U.S.A.). In parallel, a non-targeting Control Pool siRNA was used as negative control (si-CTR, D-001810-10-50). Lipofectamine RNAiMAX (Thermo Fisher Scientific, MA, U.S.A.) was employed as transfection reagent as instructed by the manufacturer. Silencing efficiency was assessed by IB. For transient expression in PD HG-SOC cells of pcDNA3-EGFP-ΔN Rac1-T17N plasmid, a construct expressing a dominant inactive form of Rac1, we used LipofectAMINE 2000 reagent (Life Technologies) following the manufacturer’s instructions. Cells transfected with the empty vectors pCDNA3 was used as control (MOCK).

### Immunofluorescence

Cells were fixed in 4% formaldehyde for 10 min at room temperature. Cells were then washed with PBS twice, permeabilized in 0.3% Triton X-100 in PBS for 5 min and blocked in PBS/0,5% BSA for 60 min at room temperature. After cells were incubated overnight at 4°C with anti-YAP (cat. #sc-376830, 1:150) (Santa Cruz Biotechnology). Next day, Alexa Fluor 488-labeled goat anti-mouse (cat. #A-11001, 1:250) (Life Technologies) was added as secondary antibodies for 2 h at room temperature. DAPI (Bio-Rad) was used for nuclear counterstain for 15 min at room temperature. Images of representative cells for each labeling condition were captured (scale bar: 50 μm, magnification 63×) with a Leica DMIRE2 deconvolution microscope equipped with a Leica DFC 350FX camera and elaborated by FW4000 deconvolution software (Leica, Wetzlar, Germany). The experiments were performed in triplicates.

### RhoC activation assay

RhoC GTP levels were assessed using a Rho-binding domain (RBD) affinity precipitation assay (Cytoskeleton, Inc.). Briefly, cells were lysed in 300 μl of ice-cold MLB lysis buffer (25 mM 4-(2-hydroxyethyl)-1-piperazineethanesulfonic acid, 150 mM NaCl, 1% Nonidet P-40, 10 mM MgCl2, 1 mM EDTA, 10% glycerol, and 0.3 mg/ml phenylmethylsulfonyl fluoride complemented with protease inhibitors and 1 nM sodium orthovanadate). Glutathione Stransferase (GST)-Rhotekin coupled to glutathione agarose was added to each tube, and samples were rotated at 4°C for 60 min. Beads were washed, and proteins were eluted in 25 μl of 2× Laemmli (Bio-Rad) reducing sample buffer by heating to 95°C for 5 min. Detection of Rho-GTP was performed by IB analysis using anti-Rho A-B-C (cat. #ab175328, 1:1000, Abcam), or specific anti-RhoC (cat. #ab180785, 1:1000, Abcam) Abs.

### Invasion assays

The cell invasive ability was determined using matrigel invasion assays. In brief, PMOV10 cells and Kuramochi cells (5 × 10^4^) depleted or not for RhoC and YAP were seeded in the upper part of Boyden chambers (BD Biosciences, NJ, U.S.A.) and stimulated in the lower part of chambers with serum-free medium alone, in the presence or absence of ET-1, treated or not with macitentan. After 24 h, the invading cells were visualized using a Diff-Quick kit (Dade Behring, IL, U.S.A.) and detected under a ZOE Fluorescent Cell Imager (Bio-Rad). Invading cells were counted using the ImageJ program.

### Collagen gel contraction assay

Collagen gel was prepared according to the manufacturer’s protocol. Collagen solution was neutralized by adding of 12 µl Ac. Acetic 0,1% and 7 µl of 1 M NaOH to 600 µl of Type-I collagen stock solution (3 mg/ml). Then, WI-38 fibroblasts (2.5 × 10^5^) suspended in 500 µl of cell culture media were added and gently mixed. The cell-laden collagen was poured into 24-well plates and incubated at 37°C for 30 min. Collagen polymerized forming disk-shaped gels that were gently detached from the edges of the culture wells. Following, the disk-shaped gels were stimulated with ET-1 and/or treated with macitentan and incubated at 37°C and 5% CO^2^ for 24 h and then photographed. The decrease of the surface area of the disk-shaped gels was used to quantify the degree of gel contractility that was measured by ImageJ. The experiments were performed in triplicates.

### Fluorescent gelatin degradation assay

Fluorescent gelatin degradation assay was utilized to estimate the capability of HG-SOC cells to form mature invadopodia able to degrade the ECM. In detail, coverslips were inverted on an 80 μl drop using Oregon Green gelatin 488 conjugate gelatin (Life Technologies Italia) and heated to 37°C. Coverslips were fixed in 0.5% glutaraldehyde for 15 min at 4°C, and after washing with PBS, the slides were quenched with 5 mg/ml sodium borohydride for 3 min at room temperature. Slides were sterilized with 70% ethanol and left in complete growth media for 1 h before use. HG-SOC cells silenced or not for RhoC or YAP were cultured on fluorescent gelatin (green)-coated coverslips in a 24-well plate and left to adhere. The cells were incubated for 72 h in different experimental conditions and then fixed in 4% formaldehyde for 10 min at room temperature and processed for deconvolution examinations [13]. Images of representative cells for each labeling condition were captured (scale bar: 10 μm, magnification 63×). The degradation area (% of cells/area), visualized as black spots within the fluorescent gelatin layer, was measured by ImageJ. The experiments were performed in triplicates.

### Patient-derived xenografts studies

Six- to eight-week-old female athymic nude-CD1 nu+ /nu+ mice (Envigo Laboratories, IN, U.S.A.) were housed in specific pathogen-free conditions. Experiments involving animals and their care were conducted with the consent of the IRCCS Regina Elena Cancer Institute Animal Care and Use Committee and the Italian Ministry of Health (D.lgs 26/2014, authorization number 1083/2020PR, issued 5 November 2020 by Ministero della Salute) at the Regina Elena Cancer Institute Animal Facility. Mice were maintained in a barrier facility on high‐efficiency particulate air HEPA‐filtered racks and received food and water ad libitum. The mice were housed in single cages with wood-derived bedding material with a 12 h’s light/dark cycle under controlled temperature.

HG-SOC-PDX were generated by nude mice intraperitoneal (i.p.) injection of PD HG-SOC cells (2.5 × 10^6^ in 200 μl PBS), as previously reported [17]. Upon a latency of 7 days, mice were randomly subdivided into two groups (*n* = 8), undergoing the following treatments: control (CTR; vehicle) versus macitentan (MAC; 30 mg/kg/oral daily). The control group underwent the same schedule as those mice given the active drug. Mice were monitored daily and subsequently killed when they presented signs of distress due to disease progression. Notably, during the experiments we did not observe body weight loss in the two treatment groups. Following 4 weeks, mice were killed by cervical dislocation and intraperitoneal tumor nodules were taken throughout the peritoneal cavity for *ex vivo* analysis. Values represent the mean of the number of visible metastases ± SD of eight mice in each group from two independent experiments.

### Statistical analysis

Student’s *t*-test was used for the analysis of the comparison between two groups of independent samples. Data points represent the mean and standard deviation (SD) of three independent experiments performed in triplicates for all the conditions described. The analysis of the data was conducted in GraphPad Prism v8.0 software.

## Results

### RhoC and Rac1 act as mediators of the ET-1/ET-1R axis-induced YAP activation in HG-SOC cells

**Figure 1:**
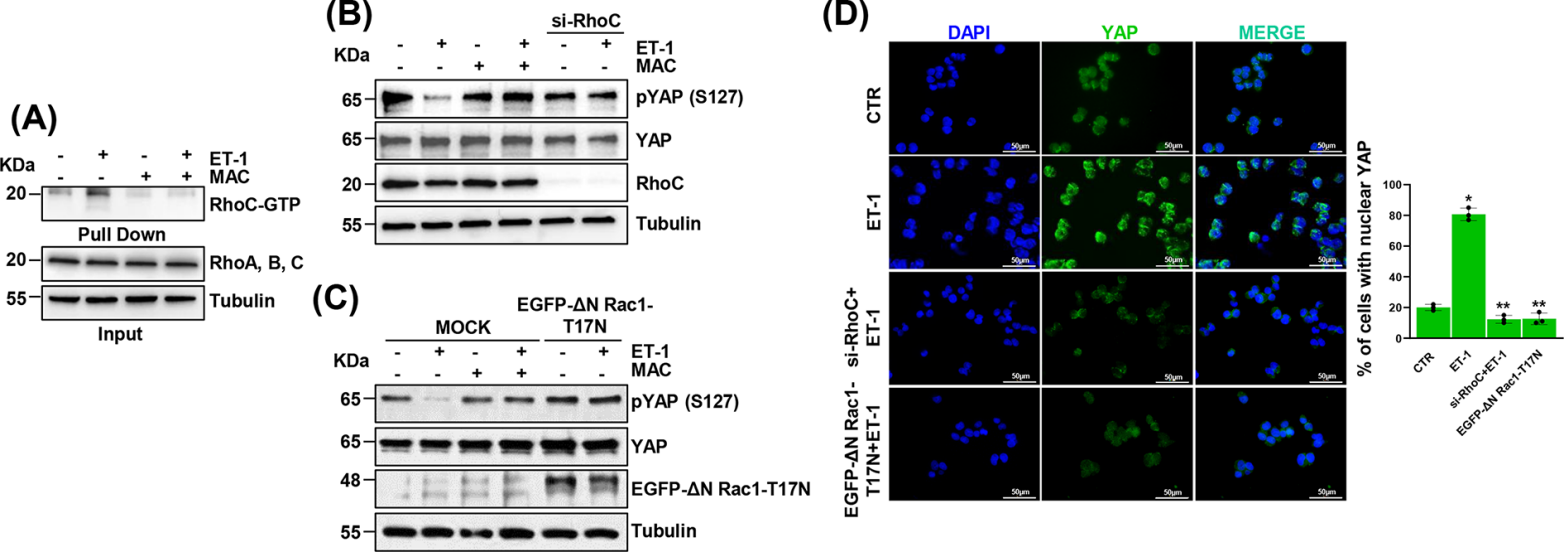
Downstream of ET-1/ET-1R axis RhoC and Rac1 GTPases mediate YAP activation in HG-SOC cells (**A**) Rhotekin beads were used to pull down RhoC-GTP from PD HG-SOC cells stimulated with ET-1 (100 nM) and/or macitentan (MAC, 1 μM) for 5 min. Pull down samples and inputs were analysed by WB for the indicated proteins. (**B,C**) Immunoblotting (IB) analysis for pYAP (S127), YAP and RhoC (**B**) and pYAP (S127), YAP and Rac1 (**C**) in total extracts of PD HG-SOC cells, silenced or not for RhoC for 72 h (**B**) or transiently transfected with EGFP- ΔN Rac1-T17N plasmid for 24 h and stimulated or not with ET-1 and/or MAC for 2 h. Tubulin was used as a loading control. Uncropped gels of Fig. A-C are shown in Supplementary Figure S3-S5. (**D**) YAP localization evaluated by immunofluorescence (IF) in PD HG-SOC cells stimulated with ET-1 and/or MAC for 2 h. Nuclei are stained in blue (DAPI). Right graph represents the percentage (%) of cells with nuclear YAP (scale bar: 50 µm, magnification 63×). Bars are means ± SD (*P<0.0002 vs. CTR, **P<0.0002 vs. ET-1; *n* = 3).

Mounting evidences emphasize the central role of the Rho subfamily of GTPases, including RhoC and RhoA, in supporting HG-SOC cell invasiveness [6–8,11–15]. Beyond the previously reported RhoA activity [11,12,17], we measured by pull-down assays the RhoC GTPase activity in response to ET-1/ET-1R axis activation, detecting a significant increase in RhoC GTPase levels upon patient-derived (PD) HG-SOC primary cells stimulation with ET-1. This effect was reversed by cell treatment with the dual ET-1R antagonist macitentan (Figure 1A). Considering that downstream of ET-1R YAP activity is heavily implicated in conferring to HG-SOC cell invasive features [16–20], and taking into account that the Rho GTPases-driven signaling may regulate YAP functions [16–19,21,23], we analysed the YAP phosphorylation status in reply to Rho-GTPases-driven signaling deactivation. In particular, we observed that RhoC depletion in PD HG-SOC primary cells and in the HG-SOC cell line, Kuramochi, interfered with the ET-1-driven YAP de-phosphorylation and activation, to an extent similar to that one produced by macitentan (Figure 1B, Supplementary Figure S1A–C and Supplementary Figure S10 and S11). Along with RhoC, also the Rho GTPase Rac1, has been reported to actively sustain the tumor cell invasive behaviour [21,24,25] and to activate YAP [26]. Thus, we thought to examine the effect generated by Rac1 inactivation on YAP phosphorylation. HG-SOC cell transfection with a construct expressing a dominant inactive form of Rac1 (EGFP-ΔN Rac1-T17N), lead to a significant increase of YAP phosphorylation, with an effect comparable to that one observed in response to cell treatment with macitentan (Figure 1C). In agreement with these findings, the immunofluorescence analysis unveiled how the ET-1-triggered YAP nuclear accumulation was lowered upon RhoC depletion or Rac1 inactivation (Figure 1D). Overall these results provide a first evidence of the signaling interlink existing between the ET-1/ET-1R/RhoC/Rac1 axis and YAP activity in HG-SOC cells, suggesting that both RhoC and Rac1 GTPases are required for the ET-1-guided YAP de-phosphorylation and resulting activation.

### YAP mediates the ET-1/ET-1R-induced invadopodia degradative ability

**Figure 2:**
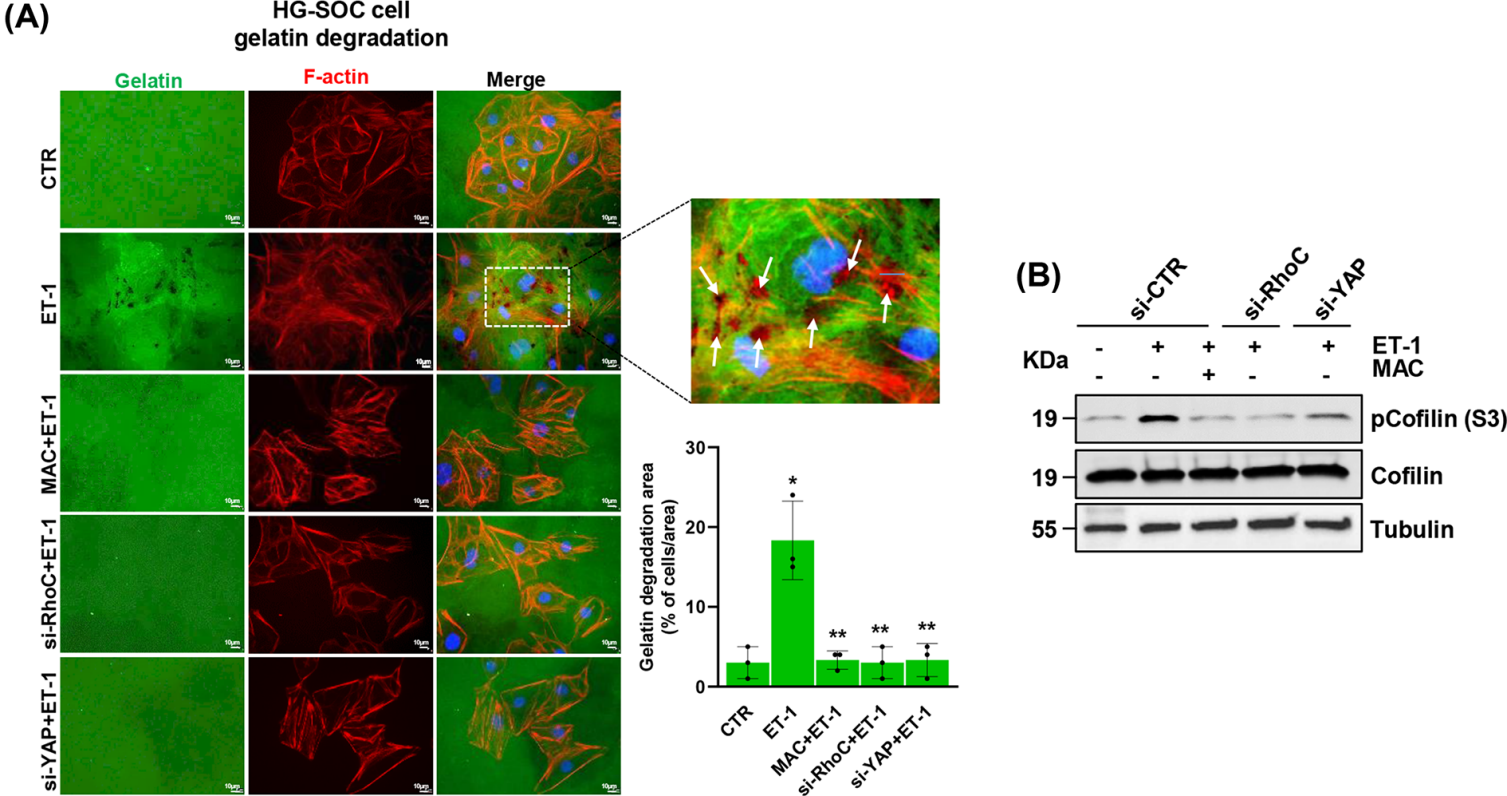
The ET-1/ET-1R/RhoC/YAP axis induces the invadopodia-mediated ECM degradation (**A**) IF analysis of Kuramochi cells silenced or not for RhoC or YAP for 72 h and stimulated or not with ET-1 and/or treated with MAC for 72 h, plated on to gelatin matrix (green). Representative images show F-actin structures (red), and nuclei (blue, DAPI). Co-localization of the gelatin degradation area (black spots) and F-actin structures is shown in the merged images (indicated by arrows) and reported as an enlarged picture. Experiments were performed in triplicates (scale bar: 10 μm, magnification 63×). Bars are means ± SD of the degradation area (% of cells/area) (*P< 0.008 vs. CTR; **P<0.009 vs. ET-1; *n* = 3). (**B**) IB analysis for pCofilin (S3) and Cofilin in total extracts of Kuramochi cells, silenced or not for RhoC or YAP for 72 h, stimulated or not with ET-1 and/or MAC for 1h. Tubulin was used as a loading control. Uncropped gels of Fig. B are shown in Supplementary Figure S6.

To establish whether YAP signaling may have a pivotal role in the ET-1-mediated invadopodia proteolytic activity and ECM degradation, we examined the ability of HG-SOC cells to produce ventral actin-rich protrusions, the invadopodia, when plated on fluorescent green gelatin and assayed for ECM degradation, in which the degradation areas appeared as black spots, characterized by the loss of fluorescence. As shown by the immunofluorescence (IF) and by the gelatin degradation area measurement, stimulation with ET-1 significantly increased the ability of HG-SOC cells to degrade. Most importantly, the punctate actin signals mostly overlap with such areas of gelatin degradation (Figure 2A). Notably, macitentan abolished the ET-1-driven invadopodia formation (Figure 2A). RhoC or YAP silencing exerted an effect similar to macitentan (Figure 2A and Supplementary Figure S1D, E).

Considering that cofilin phosphorylation at Ser3 (S3) represents a key event enabling invadopodia maturation into efficient ECM-degrading structures [6,10–15] (78), we analysed its phosphorylation status in response to ET-1/ET-1R signaling interference by macitentan, or upon RhoC or YAP silencing. Remarkably, we observed that the ET-1-driven up-regulation of cofilin phosphorylation was prevented by macitentan treatment, with an effect similar to RhoC or YAP depletion (Figure 2B and Supplementary Figure S1D,E). Altogether, these results indicate that downstream of the ET-1/ET-1R axis, the YAP signaling module, being involved in cofilin phosphorylation and consequent activation, contributes to generate protrusive forces to form active invadopodia that coordinate ECM degradation, thus representing a critical path in the ET-1-driven metastatic dissemination.

### The ET-1/ET-1R axis and YAP signaling convergence sustains HG-SOC invasion and cytoskeleton dynamics

**Figure 3:**
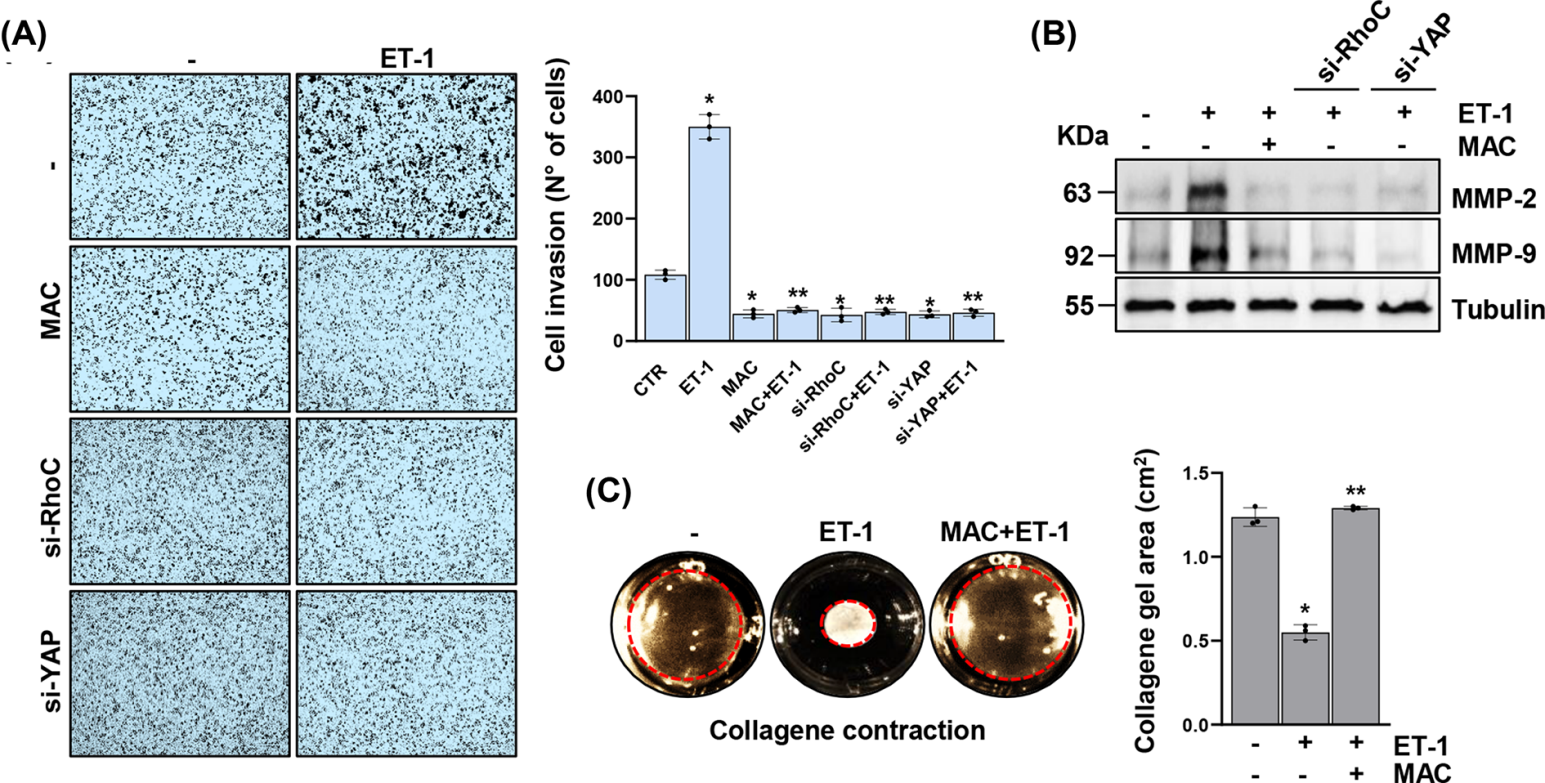
The ET-1/ET-1R-driven RhoC/YAP signaling sustains HG-SOC invasion and cytoskeleton dynamics (**A**) Invasion assay of PD HG-SOC cells silenced or not for RhoC or YAP for 72 h and stimulated or not with ET-1 and/or treated with MAC for 24 h. Representative images of invading cells were photographed (scale bar: 100 µm, magnification 20×) (*left panels*) or counted (*right graph*). Bars are means ± SD (*P<0.002 vs. CTR; **P<0.0002 vs. ET-1; *n* = 3). (**B**) IB analysis for MMP-2 and MMP-9 in total extracts of PD HG-SOC cells, silenced or not for RhoC or YAP for 72 h, stimulated or not with ET-1 and/or MAC for 24h. Tubulin was used as a loading control. Uncropped gels of Fig. B are shown in Supplementary Figure S7. (**C**) Collagen contraction assay of activated fibroblasts stimulated or not with ET-1 and/or treated with MAC for 24 h. Representative images of the collagen contraction were photographed (*left panels*). The right graph indicates the collagen gel area (cm^2^). Bars are means ± SD (*P<0.0002 vs. untreated collagen; **P<0.0002 vs. ET-1; *n* = 3).

In the attempt to delineate whether the ET-1/ET-1R/RhoC and YAP signaling interconnection may drive HG-SOC cell invasion, we monitored by transwell invasion assays the changes in the HG-SOC cell invasive pattern, observing that macitentan treatment, similarly to RhoC or YAP silencing, diminished the ET-1-boosted HG-SOC cell invasive potential (Figure 3A, Supplementary Figures S1A,C,D, E, and S2A).

Moreover, among the canonical invadopodia features there is the ability to control the proteolytic activity of well-known matrix metalloproteinases (MMP), as MMP-2 and MMP-9 [6,7,11,13]. In this regard, the immunoblotting (IB) analysis revealed the inhibition of the ET-1-mediated MMP-2 and MMP-9 activation upon macitentan treatment, or upon RhoC and YAP silencing, suggesting a role for the ET-1R/RhoC-driven YAP signaling in the induction of MMP proteolytic functions (Figure 3B and Supplementary Figure S1A,C).

Deeper insights into the cytoskeleton architecture changes are of utmost importance to understand the metastatic dissemination process. In this regard, because the ECM deposition and remodeling by activated fibroblasts, bolster tumor progression, invasion and metastasis [22,27], we examined whether the changes induced by ET-1, observed in a collagen contraction assay, had functional consequences on the contractile changes of the ECM. Collagen contraction was observed upon stimulation with ET-1 for 24 h. Notably, ET-1R blockade by macitentan inhibited the ET-1-promoted contraction abilities (Figure 3C). Altogether these findings suggest that the ET-1/ET-1R/RhoC-induced YAP signaling is implicated in the HG-SOC cytoskeleton rearrangement and invasion.

### Macitentan administration, shutting-down YAP activity, hinders the HG-SOC PDX metastatic burden

**Figure 4:**
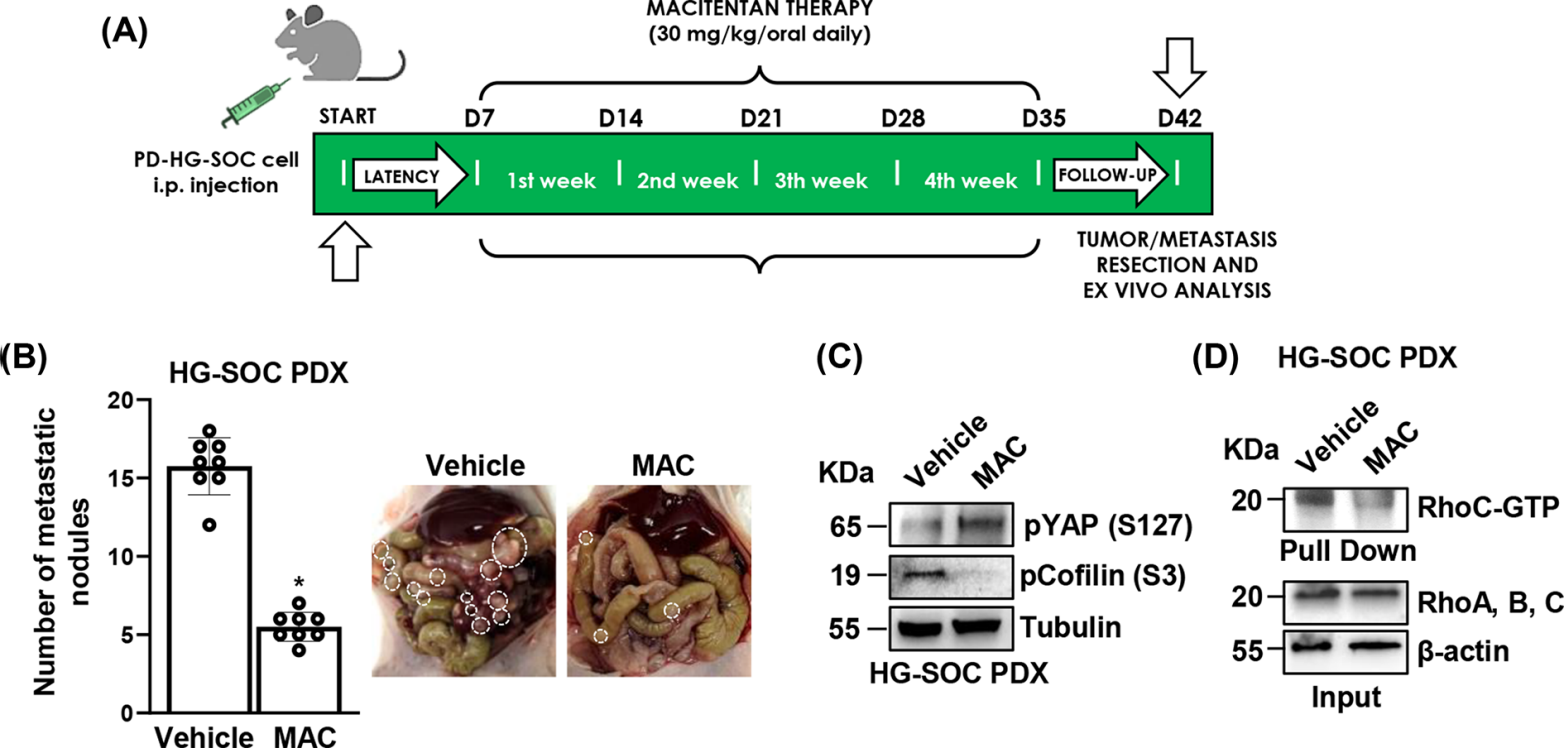
Macitentan, turning-off YAP functions, hampers the HG-SOC patient-derived xenografts metastatic potential (**A**) Treatment schedule of patient-derived HG-SOC xenografts (PDX). (**B**) The number of metastatic nodules examined at the end of the treatment. Bars are the means ± SD (*P<0.0002 vs. vehicle-treated mice; *n* = 2). *Right panels*, Representative images of the PDX metastatic load in mice treated with vehicle (*left panel*) vs. macitentan (*right panel*). The metastatic nodules are indicated by white dotted-line circles. (**C**) pYAP (S127) and pcofilin (S3) protein expression in total cell lysates of i.p. nodules were evaluated by IB analysis. Tubulin represents the loading control. (**D**) Rhotekin beads were used to pull down RhoC-GTP from total cell lysates of i.p. nodules. Pull down samples and inputs were analysed by IB for the indicated proteins. Uncropped gels of Fig. C and D are shown in Supplementary Figure S8 and S9.

Starting from the achieved *in vitro* results, to consolidate the view that to weaken the HG-SOC invasive and highly metastatic strength, treatment guidelines should be centered on the use of compounds able to hit the activity of pro-invasive and pro-metastatic signaling routes, as the YAP-driven one, that actively takes part to the invadopodia dynamic, we assessed *in vivo* the ability of macitentan to halt the RhoC/YAP-driven signal contribution to the invadopodia machinery, reducing the HG-SOC metastatic burden. To monitor the HG-SOC metastatization pattern we developed HG-SOC PDX, in which we measured the therapeutic efficacy of macitentan (30 mg/kg/oral daily) (Figure 4A). Compared with mice treated with the vehicle arm, those treated with macitentan were characterized by a remarkable reduction in the number of the metastatic lesions (Figure 4B). Along with these observations, the analysis of the protein extracts isolated from metastases emphasises how macitentan displays the ability to shut-off YAP functions, by restoring the YAP inhibitory phosphorylation on Ser127, and to interfere with the activation of cofilin, required for invadopodia maturation, as shown by the reduction on its phosphorylation at Ser3 (Figure 4C). In parallel, the RhoC GTPase pull-down assay, conducted on protein extracts isolated from the metastatic nodules as well, unveiled that macitentan curtails RhoC activation (Figure 4D). Overall these findings provide strong *in vivo* evidence of how macitentan, by suppressing the activity of RhoC/YAP at the invadopodia, greatly controls the HG-SOC metastatic colonization, featuring a potential therapeutic benefit.

## Discussion

In HG-SOC, peritoneal dissemination is intimately linked to the invadopodia formation and proteolytic activity that, unlocking the cancer cell full invasive potential, allows them to penetrate the mesothelial ECM and metastasize [4,6,7,9,11–15].

Significant advances in understanding how ET-1/ET-1R axis generates protrusive forces to form degradative structures that confer them malignant advantages have been achieved [6,7,11,13–15]. However, whether the integration of ET-1R-driven signaling with pro-oncogenic routes, as YAP-driven one, makes part to the invadopodia formation and function, demands further investigations to update the scenery of the therapeutic interventions for metastatic HG-SOC patients.

**Figure 5:**
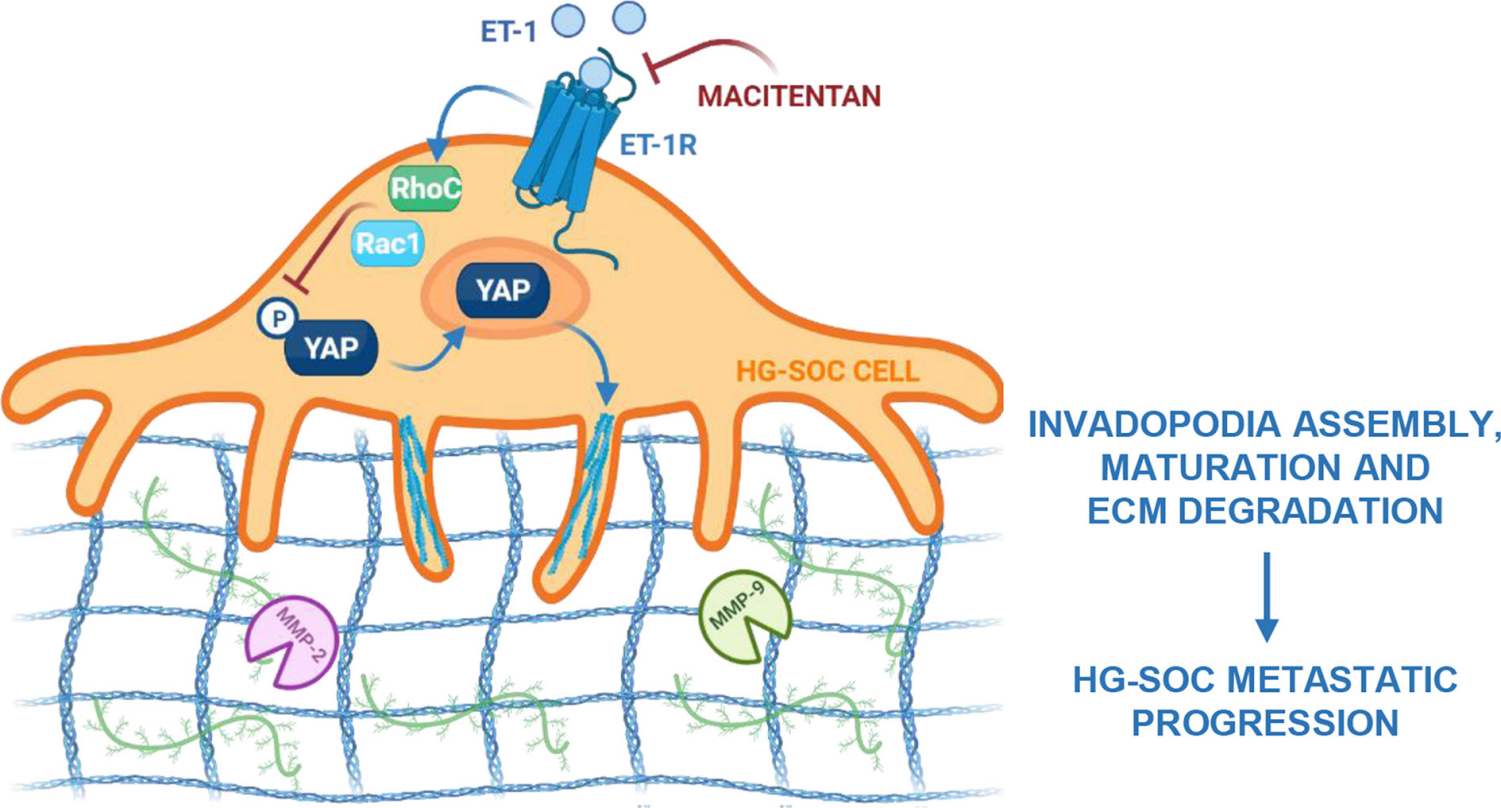
Schematic illustration of the research ET-1R activation by ET-1, inducing via RhoC and Rac1 GTPases the YAP signaling, mediates cofilin and MMP-2 and MMP-9 activities, coordinating the invadopodia-mediated ECM degradation, thus enhancing HG-SOC cell invasion and metastatization. Of clinical interest, macitentan, hindering the ET-1/ET-1R-driven RhoC/Rac1/YAP pro-invasive signaling, interferes with the HG-SOC progression. These findings highlight how ET-1R blockade, preventing the ET-1R/YAP-guided invadopodia machinery, controls the HG-SOC metastatic spread, expanding the repertoire of the therapeutic intervention for HG-SOC patients. The figure is drawn using BioRender.com.

In this perspective, this study unveiled the existence of unique signaling machinery activated under the guidance of the ET-1/ET-1R axis that, leveraging the RhoC and Rac1 GTPases, guided the YAP-driven invadopodia formation. The convergence between the ET-1R/RhoC/Rac1 and YAP signaling lead to cofilin activation and to the induction of the MMP-2 and MMP-9 proteolytic activities, sustaining invadopodia formation and maturation, enabling HG-SOC cell to acquire more aggressive traits, including the ability to disrupt the surrounding ECM and to metastasize. ET-1R blockade, breaking-down the contribution of the ET-1R/YAP-driven signaling at the invadopodia, inhibited ECM proteolysis and, consequently, the HG-SOC invasive and metastatic strength, corroborating the notion that targeting the ET-1-driven signaling may represent a valid therapeutic choice for metastatic HG-SOC patients (Figure 5).

Our observations expand previous results proving the strong clinical correlation existing between the ET-1 signaling and YAP in HG-SOC. In particular, in a cohort of HG-SOC specimens and by analysing the Cancer Genome Atlas data set, it was unveiled how the combined high expression levels of ET_A_R and YAP are associated with poor clinical outcomes in recurrent HG-SOC patients [19]. Taken together, these results prove the connection existing between the ET-1/ET-1R axis and YAP signaling activation able to reawaken the HG-SOC cell attitude to form mature invadopodia, remodel the ECM, and promote tumor metastasis.

Consistent with recent studies that highlighted the RhoA-induced YAP signaling as an important mediator of peritoneal dissemination [17], our results demonstrate the ability of RhoC and Rac1 to engage a new invadopodia regulator, YAP, delineating an unforeseen route at invadopodia by which downstream of the ET-1/ET-1R axis, the YAP-driven proteolytic signal exhibits a critical impact in controlling the invadopodia maturation, ECM degradation and HG-SOC metastatic potential.

YAP, as co-pilot of the metastatic journey, represents a central cancer vulnerability that may be exploited therapeutically [28]. Recent studies suggest that YAP may represent a master transcriptional regulator that enables tumor cells to hijack phenotypic plasticity essential for gain metastatic abilities [29]. On the basis of our findings, it is worth considering that, in response to the ET-1/ET-1R axis, YAP signaling controls the invadopodia-regulatory activity, the targeting of which may be beneficial to hamper the metastatic progression. Among the most promising molecular drugs targeting YAP, we identified ET-1R antagonists. Related to this clinical aspect, our findings emphasize the therapeutic profit associated to the use of ET-1R antagonists, that interfering with the ET-1R/YAP-dependent proteolytic signaling to invadopodia and with the associated metastatic spreading, may expand the therapeutic prospects for advanced stage HG-SOC patients.

## Clinical perspectives

- In HG-SOC the ability to generate invadopodia frequently mirrors the invasive rate of tumor cells. Thus, the identification of invadopodia regulators, along with the definition of the mechanisms directing invadopodia dynamics represents a fascinating field of study. In this perspective, defining how the ET-1/ET-1R-engaged oncogenic signaling pathways impact on the invadopodia system merits to be further explored.
- This study demonstrates how the ET-1/ET-R axis, via RhoC and Rac1 GTPases, hijacks YAP that, in turn, orchestrates invadopodia assembly and maturation, strengthening the HG-SOC pro-metastatic potential.
- Clinical significant, our findings substantiate the concept that ET-1R blockade, interfering with the signaling network of proteins that regulate the invadopodia machinery and the metastatic dissemination, embodies a potential therapeutic choice for advanced HG-SOC patients.

## Supplementary Material

Supplementary Figures S1-S11

## Data Availability

All data generated and analysed during the current study are included in this article or from the corresponding authors (A.B. or P.T.) on reasonable request. PD HG-SOC primary cells will be made available to academic researchers with material transfer agreement. All data and reagents are available from the authors upon request.

## Abbreviations

β-arr1: β-arrestin1
AIRC: Italian Foundation for Cancer Research
ECL: enhanced chemiluminescence
ECM: extracellular matrix
EMEM: Eagle’s minimum essential medium
ET-1: endothelin-1
ET-1R: endothelin-1 receptors
ET_A_R: endothelin-1 A receptor
FDA: Food and Drug Administration
HG-SOC: high-grade serous ovarian cancer
IB: immunoblotting
IF: immunofluorescence
IRB: Regina Elena Institutional Review Board
JCRB: Japanese Collection of Research Bio resources
PD: patient-derived
PDX: patient-derived xenograft
SD: standard deviation
TME: tumor microenvironment
