# Supplementary material for: YAP signaling orchestrates the endothelin-1-guided invadopodia formation in high-grade serous ovarian cancer
Source: Biosci Rep. 2024 Nov 29;44(12):BSR20241320. doi: 10.1042/BSR20241320 (PMC11609349; doi:10.1042/BSR20241320)

Supplementary Information

YAP signaling orchestrates the endothelin-1-guided invadopodia formation in high-grade serous ovarian cancer

Piera Tocci[1*], Valentina Caprara[1], Celia Roman[1], Rosanna Sestito[1], Laura Rosanò[1,2], and Anna Bagnato[1]*.

[1]Preclinical Models and New Therapeutic Agents Unit, Istituto di Ricovero e Cura a Carattere Scientifico (IRCCS), Regina Elena National Cancer Institute, Rome, Italy; [2]Institute of Molecular Biology and Pathology (IBPM), National Research Council (CNR), Rome, 00185, Italy.

*Correspondence: Anna Bagnato (annateresa.bagnato@ifo.it) or Piera Tocci (piera.tocci@ifo.it).

Supplementary Figures

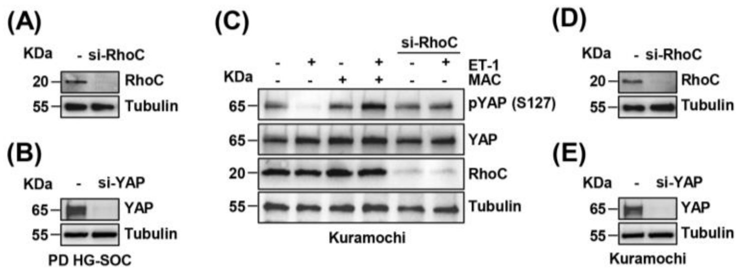

Supplementary Fig. 1. Downstream of the ET-1/ET-1R axis RhoC mediates YAP activation in HG-SOC. (A, B and D, E) RhoC and YAP protein expression in patient-derived (PD) HG-SOC cells (**A, B**) and Kuramochi HG-SOC cells (**D, E**) silenced or not for RhoC or YAP for 72 hours (h). Tubulin was used as loading control. (**C**) Immunoblotting (IB) analysis for pYAP (S127), YAP and RhoC in total extracts of Kuramochi HG-SOC cells, silenced or not for RhoC for 72 hours (h) and stimulated or not with ET-1 and/or MAC for 2 h. Tubulin was used as a loading control.

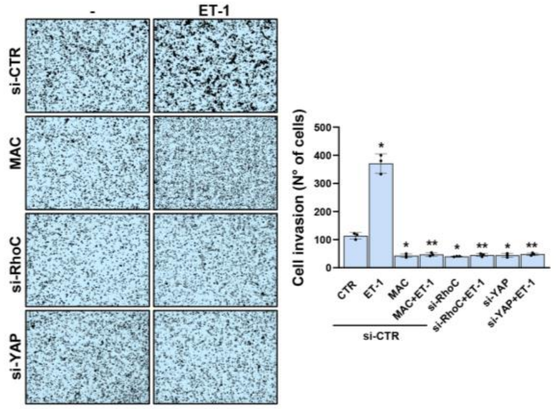

Supplementary Fig. 2. The ET-1/ET-1R and YAP signaling cooperation boosts HG-SOC cell invasion. Invasion assay of Kuramochi HG-SOC cells silenced or not for RhoC or YAP for 72 h and stimulated or not with ET-1 and/or treated with MAC for 24 h. Representative images

of invading cells were photographed (scale bar: 100 μm, magnification 20X) (*left panels*) or counted (right graph). Bars are means ± SD (*$p < 0.002$ vs. CTR; **$p < 0.0002$ vs. ET-1; $n = 3$).

Uncropped western blots

Supplementary Fig. 3. Uncropped western blots of Fig. 1A

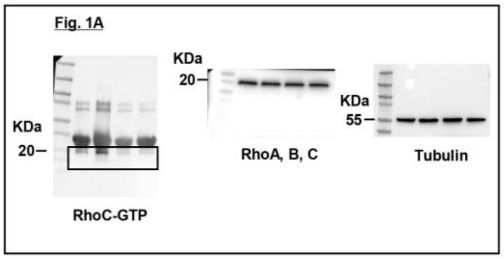

Supplementary Fig. 4. Uncropped western blots of Fig. 1B

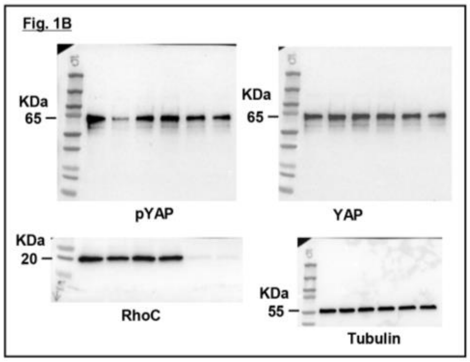

Supplementary Fig. 5. Uncropped western blots of Fig. 1C

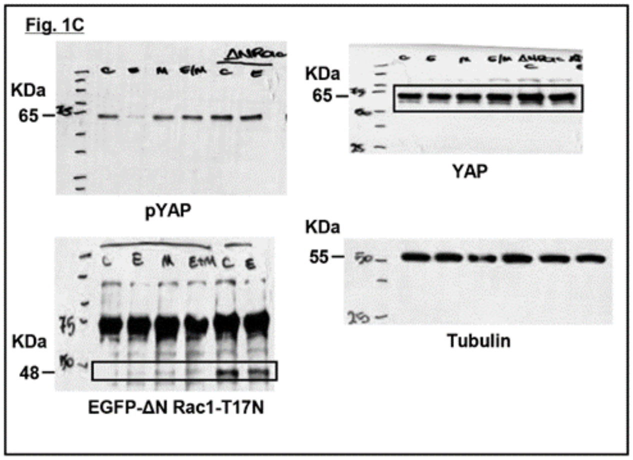

Supplementary Fig. 6. Uncropped western blots of Fig. 2B

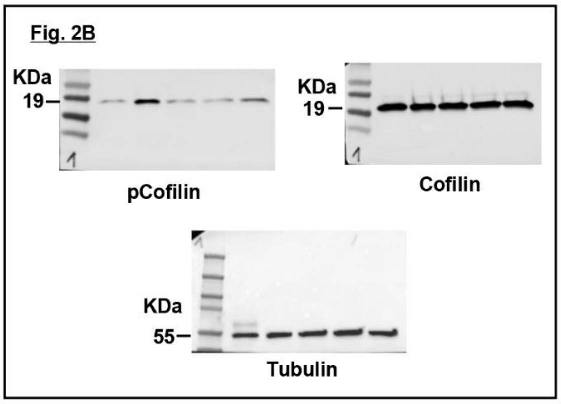

Supplementary Fig. 7. Uncropped western blots of Fig. 3B

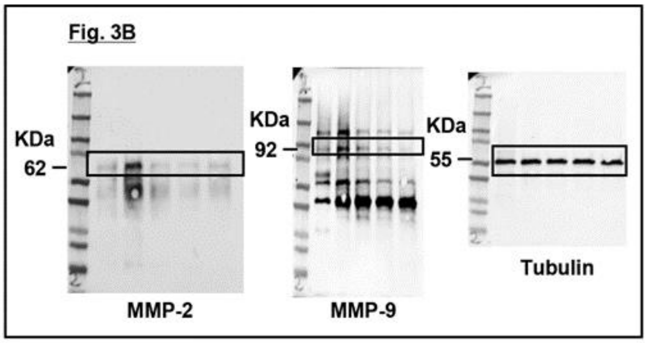

Supplementary Fig. 8. Uncropped western blots of Fig. 4C

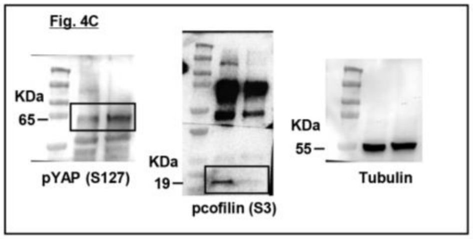

Supplementary Fig. 9. Uncropped western blots of Fig. 4D

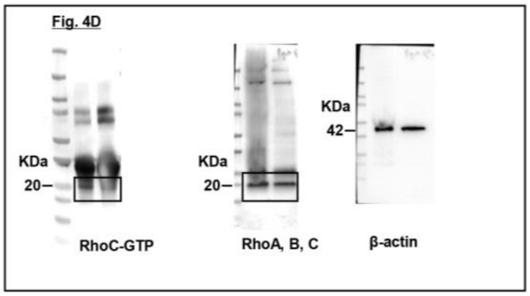

Supplementary Fig. 10. Uncropped western blots of Supplementary Fig. 1A, B and 1D, E

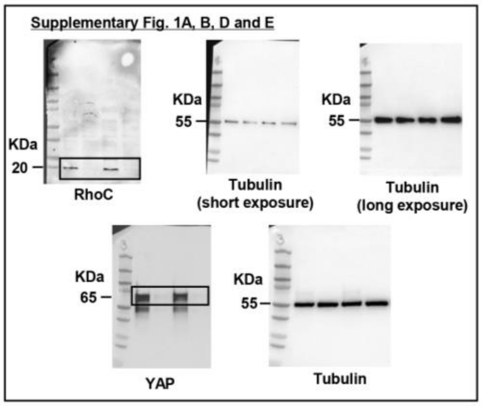

Supplementary Fig. 11. Uncropped western blots of Supplementary Fig. 1C

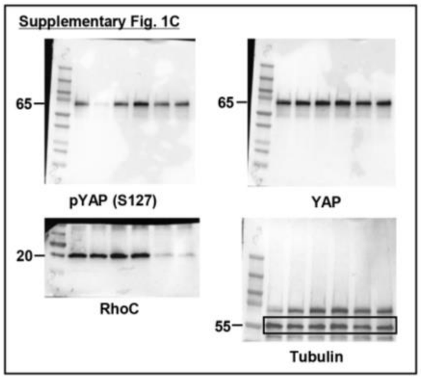

Supplement: Supplementary Figures S1-S11 [file BSR-2024-1320_supp.pdf]
